# A novel method to understand tumor cell invasion: integrating extracellular matrix mimicking layers in microfluidic chips by “selective curing”

**DOI:** 10.1007/s10544-017-0234-8

**Published:** 2017-10-17

**Authors:** H. Eslami Amirabadi, S. SahebAli, J. P. Frimat, R. Luttge, J. M. J. den Toonder

**Affiliations:** Microsystems Group, Department of Mechanical Engineering and Institute for Complex Molecular systems (ICMS), Eindhoven University of Technology, Groene Loper 15, 5612AZ Eindhoven, the Netherlands

**Keywords:** Microfabrication, 3D matrix layers, Microfluidics, Cancer cell invasion, Extracellular matrix architecture

## Abstract

A major challenge in studying tumor cell invasion into its surrounding tissue is to identify the contribution of individual factors in the tumor microenvironment (TME) to the process. One of the important elements of the TME is the fibrous extracellular matrix (ECM) which is known to influence cancer cell invasion, but exactly how remains unclear. Therefore, there is a need for new models to unravel mechanisms behind the tumor-ECM interaction. In this article, we present a new microfabrication method, called selective curing, to integrate ECM-mimicking layers between two microfluidic channels. This method enables us to study the effect of 3D matrices with controlled architecture, beyond the conventionally used hydrogels, on cancer invasion in a controlled environment. As a proof of principle, we have integrated two electrospun Polycaprolactone (PCL) matrices with different fiber diameters in one chip. We then studied the 3D migration of MDA-MB-231 breast cancer cells into the matrices under the influence of a chemotactic gradient. The results show that neither the invasion distance nor the general cell morphology is affected significantly by the difference in fiber size of these matrices. The cells however do produce longer and more protrusions in the matrix with smaller fiber size. This microfluidic system enables us to study the influence of other factors in the TME on cancer development as well as other biological applications as it provides a controlled compartmentalized environment compatible with cell culturing.

**Electronic supplementary material:**

The online version of this article (10.1007/s10544-017-0234-8) contains supplementary material, which is available to authorized users.

## Introduction

Recent figures of World Health Organization (WHO) show that cancer causes nearly 1 of 6 deaths worldwide. Prevention and management of the disease are key aspects in reducing the mortality rate. Moreover, cancer metastasis is reported to be the major cause of cancer-associated deaths (WHO 2017). Cancer metastasis consists of multiple steps. In the first step, tumor cells escape from the primary tumor and invade into the surrounding tissue until they find their way into the vascular system, a process called intravasation. The tumor cells can then spread in the circulatory system throughout the body. As soon as intravasation occurs, the control over the spread of cancer cells is extremely challenging. This is why many studies are devoted to better understand the mechanisms of cancer cell invasion during the first step in order to eventually find ways to prevent metastasis (Chiang et al. 2016; Friedl and Wolf 2003).

It is now well established that, along with genetics, tumor microenvironment plays a major role in the invasion of cancer cells towards the circulation (Bussard et al. 2016; Pietras and Östman 2010). The microenvironment of a tumor consists of different cell types named stromal cells, a diversity of growth factors and cytokines, and extracellular matrix (ECM). Among these elements, ECM provides cells with biochemical and biophysical support and functions as one of the key regulators of cancer cell invasion. ECM is mainly fibrillar and present in the interstitial space of most tissues (Frantz et al. 2010; Stetler-Stevenson et al. 1993). During cancer cell invasion, ECM is remodeled continuously by the cells through the secretion of proteins such as matrix metalloproteinases (MMPs) and lysyl oxidases (LOX) (Deryugina and Quigley 2006; Levental et al. 2009). This remodeling induces changes in the stiffness of the matrix as well as the architecture of the fibers. These changes eventually facilitate the invasion of cancer cells towards the vasculature. For example, during the invasion, the stiffness of the interstitial matrix increases and microtracks with oriented collagen fibers are formed (Blackmon et al. 2016; Cukierman and Bassi 2010; Lu et al. 2012). Despite recent advances in tumor models, the bilateral interplay between the ECM and cancer cells is not fully understood. For example, it remains unknown whether the invading cells change the topography of the ECM fibers or the remodeled ECM precedes and promotes tumor cell invasion (Polacheck et al. 2013). Therefore, controlled reductionist models are needed in order to study and understand the complexity of this interaction during cancer invasion.

Due to the complexity, high cost and ethical issues associated with animal models, *in vitro* cancer invasion models are popular among scientists (Alemany-Ribes and Semino 2014; Unger et al. 2014). Transwell and wound healing assays are conventional 2D methods to assess the invasion of cancer cells in response to external stimuli, such as gradients of growth factors (Roussos et al. 2011). Although very informative, they lack many essential elements of the tumor microenvironment during invasion, for example 3D extracellular matrix. Recently, more advanced *in vitro* invasion models have been proposed that add more complexity to the tumor microenvironment (Alemany-Ribes and Semino 2014; Xu et al. 2014). Among these models are the three-dimensional open-culture methods that are widely used particularly because they provide direct access to the tumor (Polacheck et al. 2013). A number of studies, indeed, use these systems to understand the relationship between the physical properties of ECM and cancer cell invasion (Guzman et al. 2014; Lang et al. 2015; Sapudom et al. 2015; Sun et al. 2014). However, due to their open nature, the gradient of soluble factors around and within the tumor mass can hardly be controlled. Importantly, they lack circulatory vessels to mimic the intravasation process. In addition, co-culturing different cells in different matrices in proximity of each other is very limited in these systems. Microfluidic devices have instead been proposed to resolve these shortcomings. They are relatively cheap, fast to fabricate and flexible in design, and because the flow is laminar, the biochemical gradients around and/or in the tumor can be precisely controlled. Furthermore, 3D matrices can be patterned in these devices which gives the opportunity to co-culture stromal cells with cancer cells (Lee et al. 2016; Sung and Beebe 2014). In addition, relevant fluid shear forces are easily reproduced with minimal usage of the fluid (Seo et al. 2013). Almost all microfluidic tumor models use injectable hydrogels as the 3D ECM. Although hydrogels are well established 3D matrices, they have drawbacks when it comes to investigating the relationship between physical properties of ECM and cancer invasion. First, due to the gelation process, their ligand density, pore size and stiffness are often coupled. Therefore, it is challenging to study the effect of each of these factors, independently, on the invasion of cancer cells in hydrogels (Alemany-Ribes and Semino 2014; Polacheck et al. 2013). Second, they are not stable enough to be retrieved after the experiments for further analysis such as mechanical testing. Free-standing 3D matrices (called 3D matrix layers in the rest of the text), such as electrospun matrices, can be complementary ECM models especially when it comes to physical properties of the matrix (Bhardwaj and Kundu 2010; Sell et al. 2009). Using fabrication processes like electrospinning, the physical properties of the matrix can be well controlled. When combined with microfluidic chips, in which the concentration of soluble factors is controlled, electrospun matrices can provide a controlled model for cancer cell-ECM interaction. To be able to study cancer cell invasion through these matrix layers, they have to be as thick as at least ten times the cell size. Layers of this thickness, however, make the integration of such ECM layers in microfluidic chips difficult.

In this study, we have resolved this issue by developing a new microfabrication method, called “Selective Curing” to integrate 3D electrospun matrices in microfluidic chips. Selective curing is designed to be suitable for any porous matrix layer up to 200 μm thick independent of the material. In addition, the fabrication method allows us to retrieve the matrix after the experiment for further analysis. As a proof of principle, we have studied the invasion of breast cancer cell lines MDA-MB-231 into two free-standing matrices with distinct average fiber size. We have used electrospinning to fabricate Polycaprolactone (PCL) matrix layers, a frequently used synthetic polymer in tissue engineering which is electrospun easily with different fiber diameters (Cipitria et al. 2011; Domura et al. 2017; Guiro et al. 2015; Suwantong 2016). Using this system, we observe that while the invasion distance of the cells into these two matrices does not differ significantly, cells do produce longer and more protrusions in the matrix with smaller fiber size. Our microfluidic platform offers a unique combination of possibilities: the flexibility to change the matrix material and architecture, controllability of soluble factor gradients and the ability to retrieve the matrix after experiments for further analysis.

## Results and discussion

### Microfluidic chip with integrated electrospun matrices

**Fig. 1:**
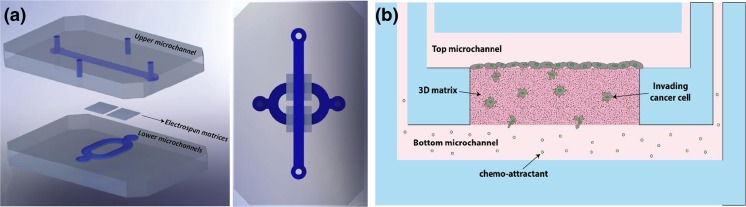
**a** Exploded and top views of the microfluidic chip. Two pieces of electrospun matrices with different properties are sandwiched between two microfluidic layers using selective curing. The top layer has a straight microfluidic channel and the bottom layer has two connected microchannels for two pieces of the matrix layers. **b** A schematic cross-sectional view of the invasion assay. The cells are seeded in the top microchannel and attracted to the bottom using a chemo-attractant

In this research, a new method, namely selective curing, to sandwich thick electrospun matrix layers between two microfluidic channels was developed (Fig. 1a). In this way, cancer cells can be seeded in one of the channels and attracted towards the other through the 3D matrix layer (Fig. 1b). The cells are more than 97% viable on the matrix inside the chip even after one week of culture (Fig. s1). To control the matrix fiber and pore size, synthetic PolyCaprolactone (PCL) fiber meshes were produced using electrospinning. Electrospinning was chosen to produce the matrix layers since it is a reliable method to fabricate fibrous meshes with diverse sizes. Varying PCL fiber and pore size was obtained by changing the settings of the electrospinning process, as detailed in the experimental section. In the following, first, the fiber and pore size of the electrospun matrices is characterized. Afterwards, the fabrication of the chip is explained, and finally the results of the invasion assay using breast cancer cell lines are reported.

### Characterization of the electrospun matrices

The porosity of the interstitial matrix influences the invasion of cancer cells, and regulates the gradient of soluble factors around the tumor mass. Therefore, it is important to characterize our ECM-mimicking layer to be able to 1. explain the invasive behaviour of the cells as a function of matrix architecture and 2. understand how effective the gradient of chemokines remains during chemotaxis. In the next sections, first the fiber and pore size of the fibrous matrix are characterized, and then the permeability of the matrix to the growth factors used in the experiments (i.e. epidermal growth factor, EGF) is evaluated.

#### Characterization of the fiber and pore size

**Fig. 2:**
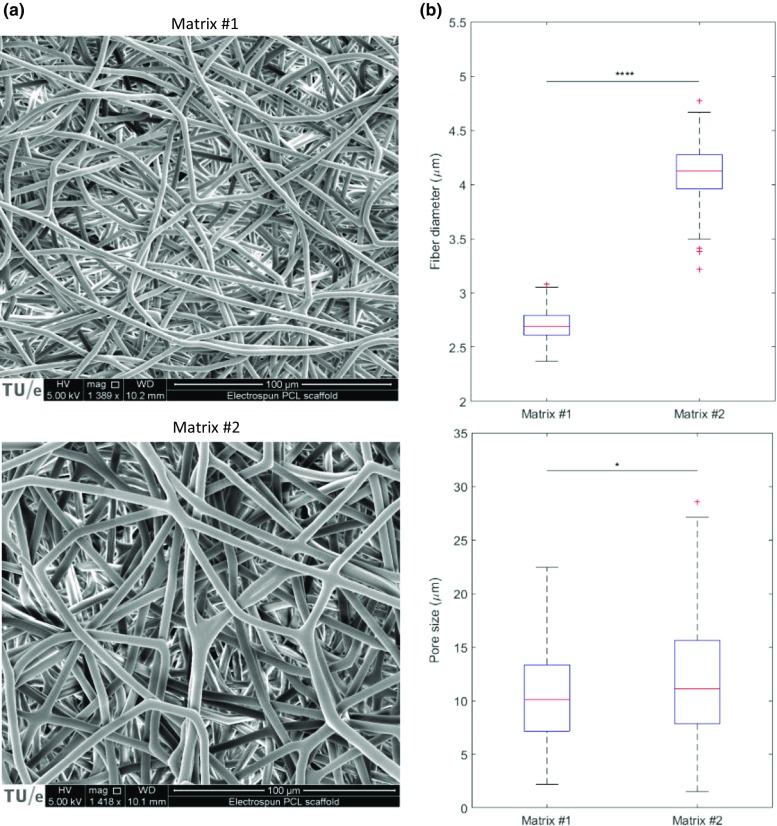
**a** Scanning electron micrographs of matrix #1 and matrix #2. **b** Box plots of the measured fiber diameter and pore size in the two matrices. The box plots divide each distribution into four sections each of which contain 25% of the data. The red lines demonstrate the medians of the distribution. The diameters of the fibers in matrix #1 and #2 are 2.7 μm and 4.1 μm, respectively (*N* = 3 independent images, *p* < 0.0001 using two sample t-test). The median pore sizes of the matrices are 10.1 μm and 11.1 μm (N = 3 independent images, *p* = 0.039 using Mann-Whitney U test). Although the difference in the median pore size is small, the spread in the pore size is larger in matrix #2

Figure 2a shows scanning electron microscopy (SEM) images of the two PCL matrices used in this study. The average fiber diameters in matrix #1 and #2 are 2.7 ± 0.1 μm and 4.1 ± 0.4 μm, respectively (Fig. 2a). However, the pore size of the matrices does not differ much (Fig. 2b), except that matrix #2 has a longer right tail in the distribution. The medians of the measured pore size in matrix #1 and #2 are 10.1 μm and 11.1 μm. The pore sizes were measured by fitting the largest possible circle in the empty space between the fibers. Although this method is subjective and thus prone to errors, the results (Fig. 2b) can help us to better understand the observations in the invasion assay. Furthermore, the average size of MDA-MB-231 cells in the rounded state is around 16 μm (Liu et al. 2015). In combination with Fig. 2, it shows that the majority of the cells are likely to stay on top of the matrix when seeded which was confirmed with control experiments too (Fig. s2).

#### Gradient of chemoattractant in the matrices

In order to calculate the chemotactic gradient along the thickness of each matrix, we need to know the permeability of the matrices to EGF. Therefore, changes in the fluorescence intensity of FITC dextran (with similar molecular size as the EGF molecules) diffusing into the matrix were measured. In parallel, a computational model was made in COMSOL Multiphysics using the geometry of the chip with varying permeability of the matrix. In this model, the time dependent diffusion equation was assumed to be the governing equation in different domains within the chip:

$$ \frac{\partial C}{\partial t}+{D}_i\Delta C=0 $$

where *C*, t and *D*_*i*_ are the concentration, time and diffusion coefficient in each domain, respectively. Inside the microchannels, the diffusion coefficient of dextran molecules in water *D*_*w*_ = 1.66 × 10^−10^ *m* ^2^/*s* was assumed (Heo et al. 2010). The diffusion coefficient in the matrix, *D*_*M*_, follows:

$$ {D}_M={RD}_w $$

By definition, *R* is the ratio of the diffusion coefficients in the matrix and in water. The computational model was run for different values of *R* and the resulting graphs were fit on the experimental data (diffusion of dextran molecules in the matrices). The results (Fig. s3) show that R can be in the range of 0.03 to 0.1 in matrix #1 and 0.07 to 0.2 in matrix #2. This data is reproduced for the center of the matrix in Fig. s4. As shown in this figure, the concentration difference between the top and bottom of both matrices can decrease more than 50% within the first hour of the experiment. This implies that the chemotactic gradient acts as a driving force for the cells to migrate through the matrix only during the first 1–2 h, unless the chemoattractant is refreshed. Indeed one of the advantages of microfluidic systems is the ability to measure such permeabilities since enough control over the concentration of soluble factors can be achieved. In addition, the calculated time scale indicates that the medium in the system should be refreshed to maintain a stable chemotactic gradient. In the current study, the media were refreshed 2 and 14 h after the cells attached to the bottom of the microchannels. The duration of refreshing flow is less than 10 s each time (2 times in total) and the duration of the experiment is 24 h. It is known that we need hours of exposure, regardless of the interstitial fluid flow rate, to have effective shear stress from the interstitial flow (Polacheck et al. 2014). Therefore we can assume that the interstitial flow plays no role in the migration of cancer cells. Refreshing the media can be automated by connecting a perfusion system to the microfluidic chips, which adds to the benefits of using microfluidic devices in maintaining conditions within or around tumors.

### Fabrication of the chip

**Fig. 3:**
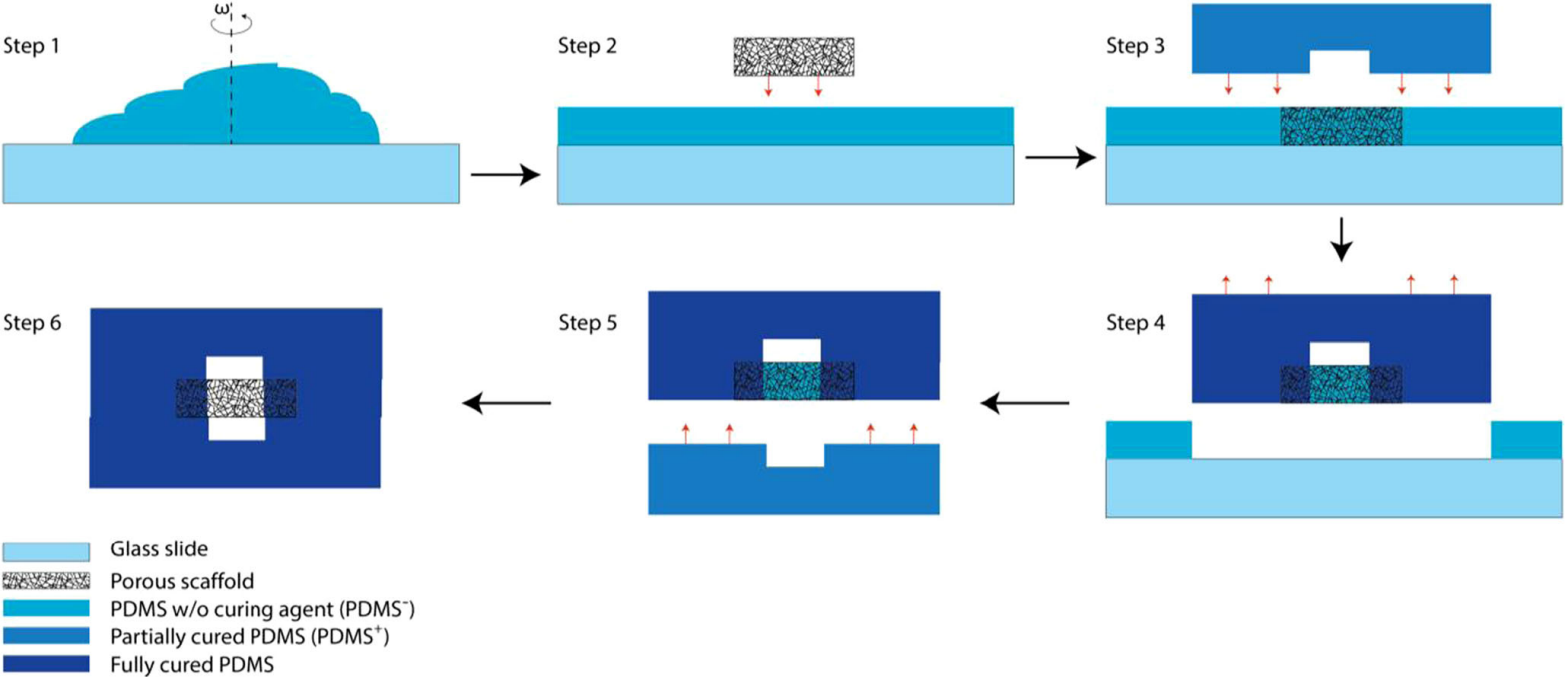
Fabrication method “selective curing”. Electrospun matrices of up to 200 μm thick can be integrated between two microfluidic channels using this fabrication method. First, liquid PDMS w/o curing agent (PDMS^−^) is spin coated on a glass slide (step 1) and pieces of the electrospun matrix are immersed in the resulting PDMS^−^ film (step 2). Then a partially cured PDMS layer (top layer, PDMS^+^) with microchannel features is put on the matrix at the desired location and cured (step 3). During this process, curing agent molecules diffuse from this layer to the liquid PDMS and solidify it. A second layer of PDMS (bottom layer) is then partially cured and aligned with the first layer (steps 4 and 5). The PDMS absorbed in the matrix is not solidified where it is in contact with microchannels. Therefore, it can be removed by flowing a solvent (IPA) across the matrix (step 6)

Fabrication of microfluidic chips with integrated porous membranes of up to 20 μm is well-established in the literature (Chueh et al. 2007; Huh et al. 2010). However, in order to study cancer cell invasion, thicker matrix layers are required, typically with a thickness in the order of at least ten times the cell size. As the thickness of the porous layer increases, it becomes challenging to fill the gap created between the device layers and around the porous layer. In this study, selective curing was developed to integrate electrospun matrices (as thick as 200 μm) between two microfluidic channels. We call the fabrication method “selective curing”. Figure 3 shows different steps of this method. The basic principle of the method is the diffusion of the curing agent (CA) molecules from PDMS with CA (PDMS^+^) to PDMS without CA (PDMS^−^). The former is partially cured to keep a fraction of CA molecules free to move. During the fabrication, first, pieces of the electrospun matrix with thickness *t* are immersed in a spin coated layer of PDMS^−^. Then a partially cured PDMS^+^ microchannel (top layer) is aligned to the matrix pieces. Over time, CA molecules diffuse from this layer to the PDMS^−^ and solidify it. The distance (*d*) over which these molecules can over time penetrate into the PDMS^−^ is determined by the concentration of the CA and also the level to which the PDMS^+^ is cured initially. In our fabrication, we have tuned the level that PDMS^+^ is cured to approximately have *d* = *t*. A second layer of microchannel (bottom layer) is then partially cured and aligned with the first layer. The PDMS absorbed in the matrix is solidified everywhere except where the microchannels cross each other, because the PDMS^+^ layer is not in contact with PDMS^−^ inside the matrix layer at that location, as shown in Fig. 3. It can therefore be removed by flowing a solvent (IPA) across the matrix. The result of this process is an approximately 2 mm by 2 mm PDMS free matrix between the upper and lower microchannels.

It must be noticed that if the film of PDMS^−^ is too thin, a gap around the matrix appears which in consequence leaves the top and bottom microchannels connected to each other. On the other hand, if it is too thick, the extra PDMS^−^ may leak to the microchannels. Therefore, the thickness of the PDMS^−^ layer has to be optimized for the matrices under study.

This fabrication method results in a leakage free chip in which thick porous membranes of up to 200 μm can be integrated. Additionally, one of the unique features of this fabrication method is that the top and bottom layers can be separated from each other after cell culture experiments without damaging the matrix layer. This is specifically useful since retrieving part of the matrix under study makes different biomolecular and biomechanical analytic techniques, such as secretome proteomics, PCR and stiffness testing, possible. Furthermore, this fabrication method can be extended to other self-standing matrices with different geometry and/or material. This flexibility provides a great opportunity to test other ECM forms rather than hydrogels. For example, in the future also (electrospun) collagen layers could be integrated, offering the possibility of direct imaging of cells as well as allowing for remodeling of the matrix by the tumor cells, towards an even better representation of the *in vivo* situation. Finally, matrices with different properties can be integrated in one chip which increases the throughput of the system.

### Morphology of invading MDA-MB-231 cancer cells inside the electrospun matrices

**Fig. 4:**
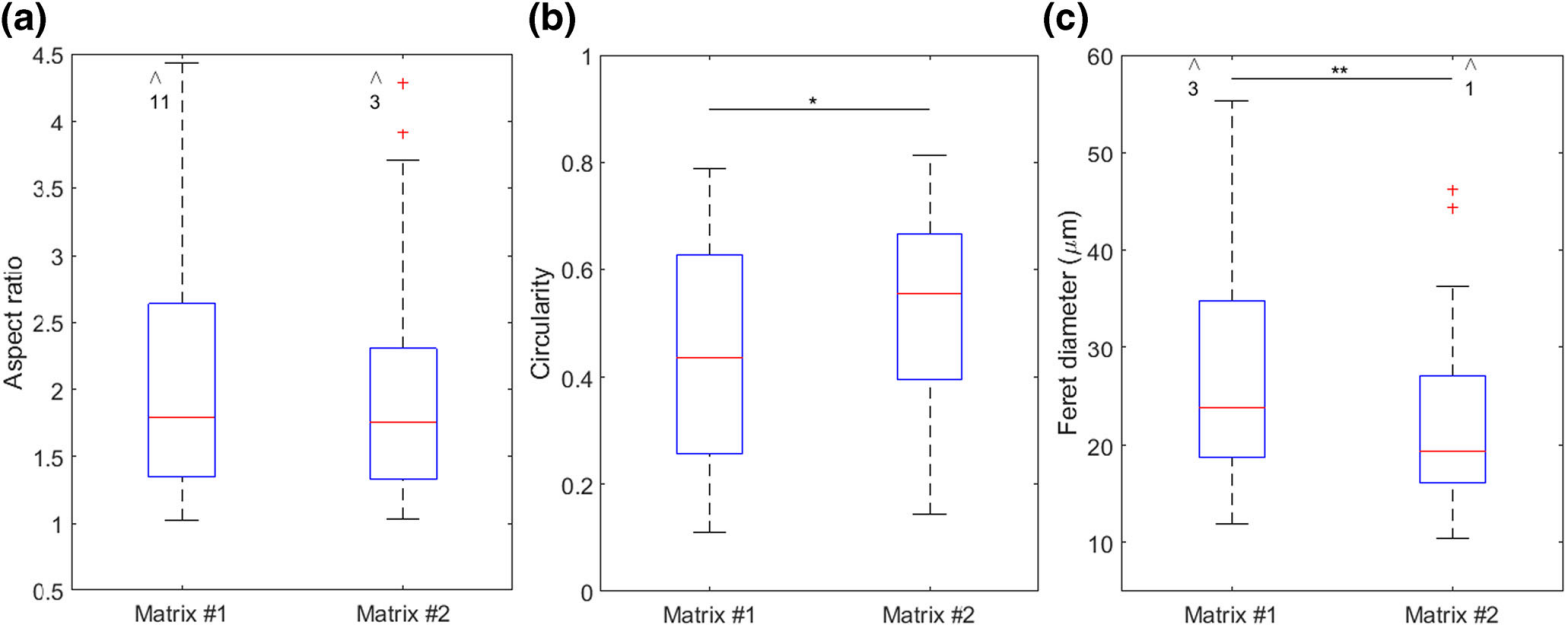
Differences in the shape of the cells invading in matrix #1 and #2 (*N* ≥ 69) in 2 and 3 independent experiments, respectively. **a** Aspect ratio (AR) of the cells; an ellipse was fit to the cells, the major axis of the ellipse divided by the minor axis results in the aspect ratio of the cells. There is more variation in the shape of cells in matrix #1 than matrix #2 with respect to AR although the average is not significantly different (~1.8). **b** Circularity of the cells; it is defined as the ratio between the area and the perimeter of the cells. The values of circularity in matrix #1 and #2 are 0.44 and 0.56, respectively. (*P* = 0.044 using Mann-Whitney U test) (**c**) The length of the longest straight line possible in the cells, their Feret diameter is 19.4 μm and 23.8 μm in matrix #1 and #2, respectively. (*P* = 0.0034 using Mann-Whitney U test) The arrows and numbers on top of the graphs demonstrate the number of outliers out of range of the axes

After carrying out the invasion assay (see Fig. 1b), the matrices were retrieved from the chips, the cytoskeleton of the cells was stained and the matrices were sectioned in 20 μm thick slices using cryosectioning. Confocal fluorescence microscopy was performed on individual cells that penetrated into the matrices and z-projected images from the confocal slices were analyzed (Fig. s5). To be able to compare the invasion phenotype of the cells, the morphology of the cells with respect to their polarity and protrusions were analyzed. Measures used are aspect ratio, circularity and Feret diameter (explained below). The results are shown in Fig. 4.

Aspect ratio (AR) of the cells is calculated by fitting an ellipse to the shape of each cell. The ratio between the major and minor axes of the ellipse gives AR for that cell. The more elongated morphology, e.g. higher AR, corresponds to a more mesenchymal type of migration, which is characteristic for MDA-MB-231 cancer cells (Wolf et al. 2003), In matrix #1, more variation in AR is observed, especially a long right tail of the AR distribution is seen, corresponding to a more stretched phenotype. However, no significant difference in the average aspect ratio of the cells in matrix #1 and #2 is observed (Fig. 4a). This implies that the difference in the fiber diameter of the matrices (while having the average pore size approximately constant) does not result in distinctly different invasive AR of the cells. This is in contrast to previous studies which report that the AR of invading cells depends on the fiber diameter, when a nano-fibrous matrix is used, but not on the pore size of the matrix (Sapudom et al. 2015). The contrast may be due to the different range of the fiber diameter or higher stiffness of the matrix in this study. Indeed in their study, Sapudom et al. found no significant difference in the spreading of the cells when the fiber diameter increased from 700 nm to 850 nm, whereas in smaller fiber diameters this difference was found to be significant. In addition, the stiffness of the hydrogels in the aforementioned study is lower than 1 kPa while the stiffness of PCL is larger than 1 MPa. It is also known that stiffness of the matrix affects cell morphology (Kumar and Weaver 2009).This indicates that for relatively large stiffness of the matrix, the changes in the fiber diameter in our study may still be too small to affect the AR of the cells. However, the current model system is still useful, the same way as for example Transwell assays, since it can provide insight on basic mechanisms behind cancer cell invasion in 3D.

Furthermore, the cellular protrusions were investigated by calculating the circularity of the cells. Circularity is a measure of how much the shape of a cell deviates from a perfect circle, for which the circularity is 1. Therefore, in addition to the eccentricity of the cell, circularity accounts for the length and the number of cell protrusions. A lower number for circularity indicates a more stretched shape and/or longer/larger number of protrusions. As seen in Fig. 4b, this number is lower for the cells in matrix #1 (0.44 vs. 0.56 in matrix #2). Considering no significant difference in AR, this result reveals that cells produce more protrusions in matrix #1 than in matrix #2. Calculating the Feret diameter of the cells in both matrices confirms this statement (Fig. 4c). The Feret diameter is the longest straight line between two points on the periphery of a cell. Larger Feret diameter corresponds to longer extensions from the cells, i.e. protrusions. Cells in matrix #1 clearly make longer protrusions, in many cases as long pseudopodia (Fig. s5). Pseudopodia are long tubular F-actin rich protrusions that are formed at the leading edge of migrating cells. Cells generate traction forces in the direction of migration using these structures (Wolf et al. 2007). The presence of longer pseudopodia in matrix #1 may suggest that the cells need larger traction force to be able to invade into the matrix. This may imply that the average pore size of matrix #1 is smaller than matrix #2. However, it may also be interpreted as a mechanism to increase cell-ECM contact area. In other words, since the surface area for the cell-ECM adhesion is smaller in matrix #1 (considering constant pore size and smaller fiber diameter), cells produce longer pseudopodia to provide more surface area for the same traction force. Further investigation is needed to study this phenomenon in more detail.

### Invasion of MDA-MB-231 cancer cells into electrospun matrices

**Fig. 5:**
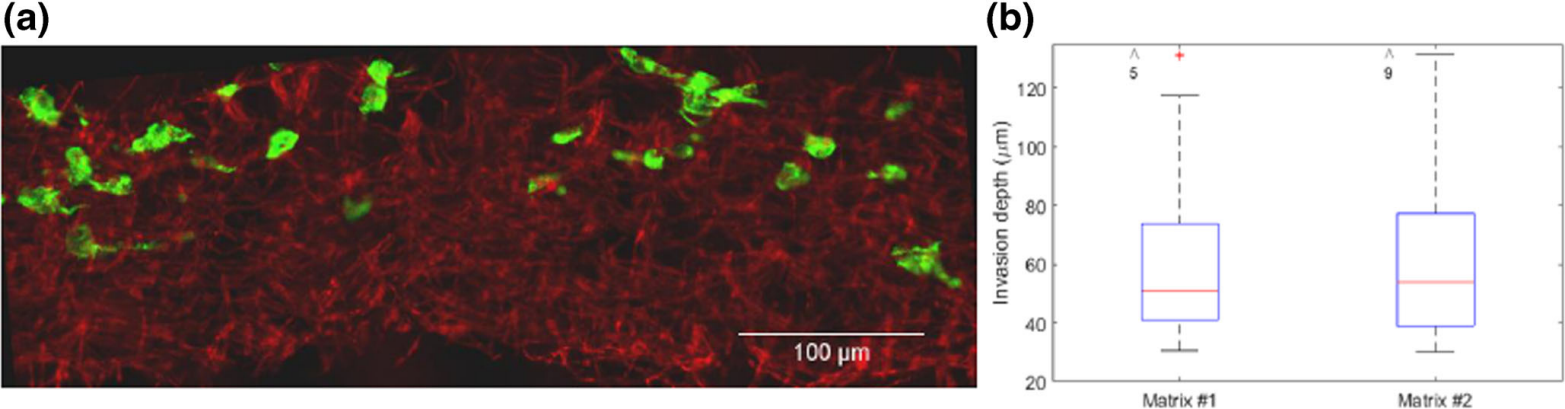
**a** A 20 μm thick cross section of an isolated electrospun matrix from the microfluidic chip; Red is the autofluorescence of the PCL matrix with filtered emission light of wavelengths below 450 nm and green is the cytoskeleton of MDA-MB-231 cancer cells. The cells are initially seeded on top and attracted towards the bottom into the matrix. **b** Invasion distance of MDA-MB-231 cancer cells (from the top) after 24 h in matrix #1 and #2. The data (*N* ≥ 147 cells from *N* ≥ 26 slices) was pooled from 3 independent experiments. The medians of invasion distance are 51 μm and 54 μm for matrix #1 and #2, respectively. The arrows and numbers on top of the graph demonstrate the number of outliers out of range of the axis

After isolation of the matrix from the chip and slicing it to 20 μm sections, fluorescence images of the matrix and (cytoskeleton of) the cells were taken. Since PCL fibers are autofluorescent above 415 nm, an open fluorescence filter above 415 nm was used to visualize the matrix. An example of such an image is shown in Fig. 5a. MDA-MB-231 cells were seeded on top of the matrix and then attracted towards the bottom, as illustrated in Fig. 1b. As seen in Fig. 5a, most cells undergo single cell migration, which is a characteristic of MDA-MB-231 cells (Volakis et al. 2014). Furthermore, Fig. 5b compares the invasion depth of the cells into matrix #1 and #2 after 24 h. Only the cells that have migrated more than two cell bodies (>30 μm) were included in the analysis. The results show that there is no significant difference between the average invasion distances of the cells in the two tested matrices although the maximum invasion distance seems to be larger in matrix #2 (equal to the thickness of the matrix). This indicates that the differences in pore size and fiber diameter between matrix #1 and #2 do not influence the infiltration depth of MDA-MB-231 cells (even though these differences affect the cell morphology, as shown in Fig. 4). Furthermore, the intrinsic orientation of the electrospun fibers can influence the invasion distance of the cells. During the electrospinning process, fibers are deposited on top of each other and are therefore aligned parallel to the collector plane. Consequently, on average the direction of migration is perpendicular to the orientation of fibers (Fig. s6). Considering that the cells cannot remodel the PCL fibers, cancer cell invasion may be hindered in our system since *in vivo* cancer cells can reorient collagen fibers parallel to the direction of migration, which facilitates invasion *in vivo* (Cukierman and Bassi 2010; Provenzano et al. 2006). Our PCL matrix still gives valuable information on the migration capacity of the tumor cells, but the intrinsic orientation of the fibers in electrospun matrices has to be taken into account in future platforms using these matrices.

## Materials and methods

### Electrospinning PCL matrices

Solutions of Polycaprolactone (Corbion Purac Biomaterials, the Netherlands) in 1,1,1,3,3,3-Hexafluoro-2-propanol (HFIP, Sigma Aldrich, the Netherlands) were made. The concentrations and the electrospinning settings for both matrices are shown in Table 1 (EC-CLI electrospinning apparatus, IME Technologies, the Netherlands). The average thickness of matrices #1 and #2 were 130 μm and 175 μm respectively.

**Table 1:** Electrospinning conditions for matrix #1 and #2

	Concentration (% *w*/w)	Voltage difference (kV)	Distance (mm)	Flow rate (μl/min)	Collector spinning speed (rpm)	Nozzle scanning speed (mm/s)	Temperature/ humidity (°C/%)
Matrix #1	12	26	270	10	500	12	23/30
Matrix #2	13,5	24	220	20	100	12	23/30

### Fabrication of the microfluidic chip

The fabrication process of the microfluidic chip is shown in Fig. 3. The process is explained here step by step:

- Step 1: Glass microscope slides were treated with soap and water and left to dry. Base silicon elastomer without curing agent PDMS^−^ (Dow Corning, Germany) was spin coated on the slides in two steps: at 500 rpm for 15 s and at 900 rpm for 1 min.
- Step 2: Electrospun matrix layers with a surface area of approximately 5 mm by 4 mm were cut and applied next to each other on the PDMS^−^ film.
- Step 3: To fabricate a mold for PDMS microchannels, photolithography was used. SU8–2150 (Micro resist technologies, Germany) was spin coated on a silicon wafer (Si-Mat, Germany) in two steps: at 500 rpm for 15 s and subsequently at 1500 rpm for 45 s. Then, the wafers were soft baked at 65^°^ *C* for 9 min and at 95^°^ *C* for 110 min. The wafer then was covered with a photomask (with the desired microchannel patterns) and exposed uniformly with the intensity of 13.6 ± 1 *mJ*/*cm* ^2^ for 37 s. The wafer was then post-baked at 65^°^ *C* for 5 min and at 95^°^ *C* for 27 min, and developed in Mr.-dev 600 (Micro resist technologies, Germany) to remove the uncured SU8 residuals. At the end, the wafer was washed thoroughly with IPA. The resulting microchannels had a thickness of 400 μm and width of 2.5 mm. 1:10 PDMS (base to curing agent) was mixed and poured on the wafer with the SU8 structures, degassed and partially cured in an oven at 65^°^ *C* for approximately 32 min (1st layer). This time may vary from batch to batch. The wafer was then left at room temperature to cool down and the PDMS was peeled off carefully. Air escape holes were punched in the PDMS, the slab was carefully aligned with the scaffolds, and left overnight to completely cure. During this process, curing agent molecules diffused to the PDMS^−^ layer and solidified it.
- Step 4: The completely cured PDMS slabs were then peeled off from the glass slide and inlets and outlets of both microchannels were punched in the slabs using 1.2 mm punchers.
- Step 5: The second PDMS layer was also partially cured slightly more than the first layer (34 min instead of 32), aligned with the first layer and left overnight at 40^°^ *C* to bond. This layer is just a bit stiffer than the first layer. It has to be soft enough to bond to the first layer well. This difference is to make the separation of the layers easier after the experiments.
- Step 6: Subsequently, the uncured PDMS (PDMS^−^ at the junction of the microchannels) was removed by flowing IPA at ~1000 *μl*/min through one of the microchannels. Female luer adapters (with the tubing cone tip cut out) were glued to the inlets and outlet using partially cured PDMS (Fig. s7).

### Diffusion experiments in the chip

Two chips and two experiments per matrix in each chip were used to assess the permeability of the matrices. FITC dextran (Sigma Aldrich, the Netherlands) with a molecular weight of 10 kDa (close to the molecular weight of EGF molecules which is approximately 6 kDa) were mixed in demineralized water to achieve a concentration of 2 mg/ml. The chips were filled with demineralized water, one of the channels was blocked, and the dextran solution was injected into the other channel using manual pipetting. Time lapse images of the light intensity from the channels with zero initial concentration of dextran were acquired for each experiment (for 15 h with 15 min intervals). The gray values of the image sequence were measured using ImageJ (National Institute of Health NIH, USA). Reference light intensity-concentration data (by measuring intensities corresponding to known concentrations in the observation points) were used to convert the gray values to concentration values.

### Simulations and data fitting

3D simulations of the matrix permeability were run in COMSOL Multiphysics. Because of chip symmetry, one quarter of the chip was simulated and no flux boundary condition was set on the symmetry planes. Initial conditions and other boundary conditions were set according to the main chemotaxis experiments. Fine and extra fine meshes were used in the channel and matrix domains, respectively. Time interval of 30 s was set as the computational time step. The simulations were performed for different diffusion ratios R and fit to the experimental data manually. After finding the diffusion ratios, the simulations were used to obtain concentration difference-time graphs inside the matrices.

### Cell culturing general

MDA-MB-231 breast cancer cells (A kind gift from Dr. Dana Mustafa, Erasmus Medical Center, Rotterdam, the Netherlands) were cultured in the culture medium which was RPMI 1640 medium (Gibco, Thermofisher Scientific, the Netherlands) supplemented with 10% (*v*/v) foetal bovine serum (FBS, Bovogen Biologicals, Australia) and 1% (v/v) penicillin-Streptomycin (Lonza, Westburg, the Netherlands) at 37^°^ *C* and 5% CO_2_. After more than 80% confluency, the cells were removed from the bottom of the culture flask using Trypsin EDTA 1× (Lonza, Westburg, the Netherlands) and centrifuged in the growth medium at 900 rpm for 5 min. The supernatant was then removed and new culture medium was added to the tube.

For chemotaxis experiments, recombinant human epidermal growth factor (EGF, Gibco, Thermo Fisher Scientific) was diluted to 50 ng/ml in the culture medium (chemotactic medium). Before chemotaxis, cells were starved in the starving medium (RMPI 1640, 1% (v/v) penicillin-Streptomycin and 0.1% bovine serum albumin) for 2 h.

### Cell culturing in the chip

PBS (Lonza, Westburg, the Netherlands) was added to the chips, and the chips were put in vacuum for 30 min to remove air trapped in cavities in the microchannels. After this step, medium exchange was done only by realizing a pressure difference (height difference) between the inlet and outlet reservoirs. In this way, better control of the flow rate and thus the chemotactic gradients could be achieved. The chips were sterilized with 70% (v/v) ethanol and washed with PBS. 50 *μg*/*ml* human plasma fibronectin (Merck Chemicals, the Netherlands) was added to the chips and incubated at 37^°^ *C* for at least 2 h. Following the incubation, chemotactic medium was added to the microchannels. After harvesting the cells, they were resuspended in the starving medium. Luers of the chips were emptied and 100 *μl* of the cell suspension of 1 × 10^6^ cells/ml was added to the inlet reservoir of the tumor channels and just a bit of liquid to the outlet to make a gentle flow (in the order of 50 *μl*/min) of cell suspension inside the channel. Different media (with and without EGF) in the microchannels were then changed 2 and 14 h after the seeding. The experiment lasted 24 h. In the case of control experiments, the experiment was for 2 h. For fixation of the cells, they were washed 2× with PBS 15 min in total and then were fixated with 3.7% paraformaldehyde (Sigma Aldrich, the Netherlands). Viability of the cells was tested using ReadyProbes cell viability imaging kit (life technologies, USA). Viability was tested after 7 days of culturing cells in the chips. 2 drops of each probe were added to the medium and were injected to the chips. The chips were incubated for 15 min and the cells were then imaged for blue (alive) and green (dead) with an EVOS FL cell imaging microscope (ThermoFisher scientific, the Netherlands). Viability of the cells was calculated as the ratio between the number of live cells to the total number of cells.

### Immunostaining and cryosectioning

After the invasion experiments, the matrix layers were harvested from the chip. First, the top channel layer was separated from the bottom one. Then, the center part of the matrix was cut out from the PDMS, making sure to obtain a sample free from PDMS since this would disturb the crysectioning performed later. The nuclei of the cells were stained with Dapi (1:500 dilution, Sigma Aldrich, the Netherlands), and their cytoskeleton was stained with Phalloidin Atto 488 (1:200 dilution, Sigma Aldrich, the Netherlands).

The stained scaffolds were then embedded in a cryosectioning glue (TissueTEK, Sakura Finetek, the Netherlands), 20 μm sections were cut at −20^°^ *C* (Using Microm HM550 cryostat microtom, Thermo Scientific, the Netherlands) and attached to Polysine slides (Thermo Scientific, the Netherlands).

### Microscopy

Mowiol 4/88 (Sigma Aldrich, the Netherlands) was applied to the sections and a cover glass was put on Mowiol to fix the sections overnight. A confocal microscope (Zeiss LSM510 META NLO, Zeiss Nederland, the Netherlands) was used to obtain all the fluorescence images. For the invasion study, a 20× objective was used. For the assessment of the morphology of the cells, a 63× oil immersion lens and confocal mode of the microscope was used. A plane interval of 3 μm was set.

All samples were sputter coated with 5 nm of gold prior to scanning electron microscopy. A scanning electron microscope (Quanta 600F ESEM, Fei, the Netherlands) with high vacuum settings and working distance of 10 mm was used.

### Data analysis

#### Cell morphology

Image stacks obtained by confocal microscopy were projected on each other (standard deviation projection type) to obtain the morphology of the cells. ImageJ was used to analyze the cell morphology. After adjusting the threshold of each image, the boundary of the cell was smoothened. Finally, the resulting shape was analyzed with respect to the aspect ratio (AR) of the fitted ellipse, its circularity and Feret diameter.

#### Invasion depth

The fluorescence images of the cells and the matrix for each section were overlayed using ImageJ. The shortest distance between each cell and top of the matrix was measured, only for the cells that had migrated more than two cell bodies (30 μm). This number was chosen to make sure only the migrated cells were captured.

#### Statistical analysis

All the data were plotted using Matlab. Kolmogorov-Smirnov was used to test the normality of the distributions. Two sample t-test and Mann-Whitney U test were used to test the significance of differences for parametric and non-parametric data, respectively.

## Conclusion

During invasion of cancer cells from the tumor into the surrounding tissue, there is a continuous cross-talk between the extracellular matrix and cancer cells. ECM is remodelled dynamically and this remodelling includes changes in both bio-chemical and bio-physical properties (Stetler-Stevenson et al. 1993). The altered ECM, in turn, influences the process of the cancer cell invasion. Limitations of current tumor models hinder us to investigate the effect of the ECM properties on cancer cell invasion independently and within a controlled microenvironment. Specifically, the influence of individual physical properties of the ECM, such as pore size, fiber diameter or elastic modulus, remains unclear. For hydrogels that are conventionally used to mimic the 3D ECM, controlling one property (e.g. pore size) without changing another (e.g. elastic modulus) is challenging. In addition, a controlled microenvironment, with precisely defined gradients of soluble factors, is not easily achieved in the current tumor models. Therefore, in this study, we have developed a novel microfluidic system that enables us to address these important issues. We have designed a new fabrication method, called selective curing, to integrate 3D ECM mimicking layers between two microfluidic channels. As a proof of concept, we have cultured invasive breast cancer cells MDA-MB-231 in the microfluidic chip. We then studied the morphology and invasion depth of the cells invading in two PCL ECM-mimicking layers under the influence of a chemotactic gradient. The matrices had similar average pore sizes but different fiber diameters. We observed that although the invasion distance and aspect ratio of the cells do not differ significantly between the matrices, the cells tend to produce more and longer protrusions in the matrix with smaller fiber size.

Selective curing is flexible and enables us to use other matrix materials, such as collagen, that more closely mimic the natural ECM from a biochemical point of view. Therefore, the effect of other properties of the matrix, such as stiffness or biochemical nature, on tumor invasion can also be studied in this system. This platform is, furthermore, suitable to study the effects of other microenvironmental factors on cancer cell invasion. For example, other cell types, e.g. endothelial cells or macrophages, can be introduced in the chip in a controlled way to investigate how these cells enhance or inhibit the invasion of cancer cells.

Up to now, we have been able to integrate matrix layers of up to 200 μm in thickness in the chip and the integration of thicker layers (especially for other applications) needs further modifications to the fabrication process. Moreover, it is important to notice that due to the electrospinning process, which is only one of the methods available to create ECM-mimicking layers, an intrinsic alignment is introduced to the matrix that is perpendicular to the chemotactic gradient in our experiments (see Fig. s6). This may impede the cell migration, however using such matrices is still useful to understand invasion mechanisms of cancer cells. To improve the long-term experimental conditions, a perfusion system can be connected to the system to maintain the relevant gradients for the complete duration of the experiments.

## Electronic supplementary material

ESM 1 (DOCX 7613 kb)
