# Supplementary material for: A novel method to understand tumor cell invasion: integrating extracellular matrix mimicking layers in microfluidic chips by “selective curing”
Source: Biomed Microdevices. 2017 Oct 17;19(4):92. doi: 10.1007/s10544-017-0234-8 (PMC5644704; doi:10.1007/s10544-017-0234-8)
Supplement: Supplementary file 1 — (DOCX 7613 kb) [file 10544_2017_234_MOESM1_ESM.docx]

A novel method to understand tumor cell invasion: integrating extracellular matrix mimicking layers in microfluidic chips by “Selective Curing”

**H. Eslami Amirabadi ∙ S. SahebAli ∙ J.P. Frimat ∙ R. Luttge ∙ J.M.J. den Toonder**^[[1]](#footnote-1)^

Supplementary information


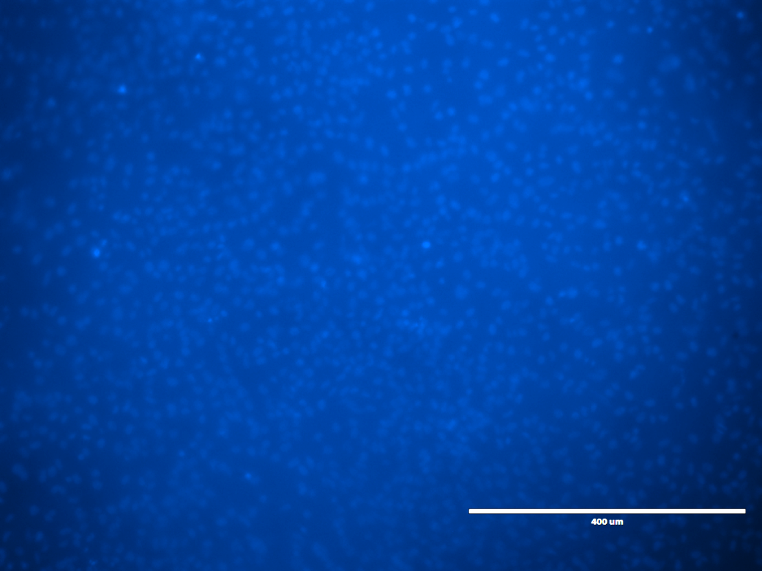

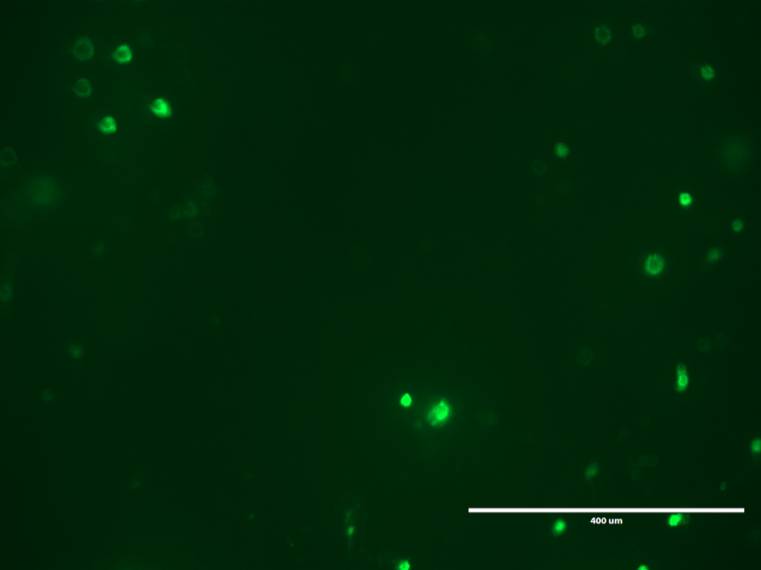


**Fig. s1** Viability of MDA-MB-231 cancer cells on an electrospun matrix inside the microfluidic chip after one week of culture; Blue and green show live and dead cells, respectively. The live cells/dead cells ratio is more than 97%. The scale bars are 400 µm.


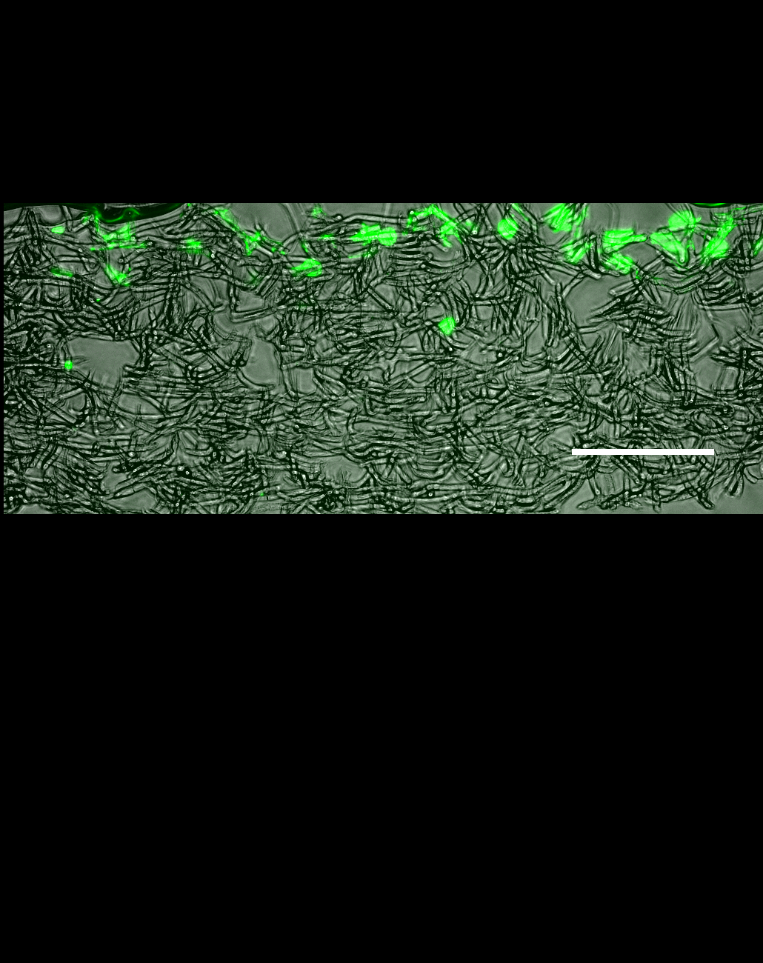


**Fig. s2** Cross section of matrix #2 in control experiments to demonstrate that the cells stay on top at the time of seeding. An image of the cells (stained with green with Phalloidin) is overlayed with a bright field image of the section. Almost all the cells stay within 30 µm from the top. Scale bar is 100 µm.


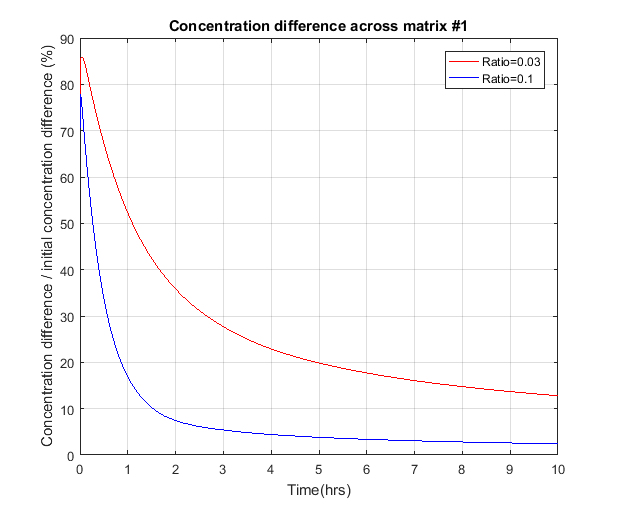

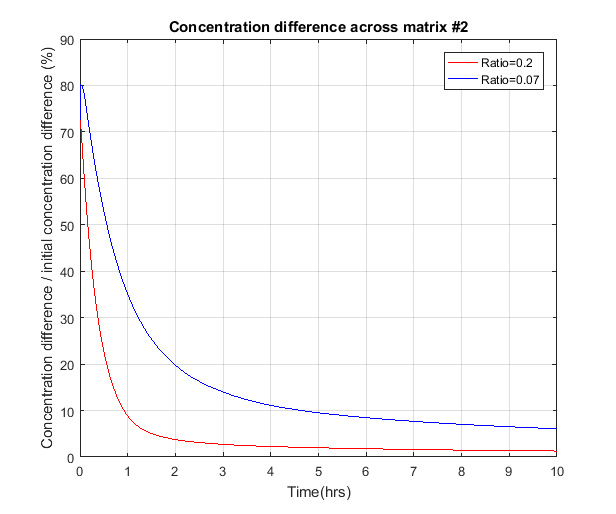


(b)

(a)

**Fig. s4** Decay in the gradient of the chemoattractant in matrix #1 (a) and #2 (b). After finding R for each experimental data set, simulations with corresponding R’s were run to realize the gradient decay. Different line graphs are for different chips. In all cases, the gradient diminishes 50% within the first hour.


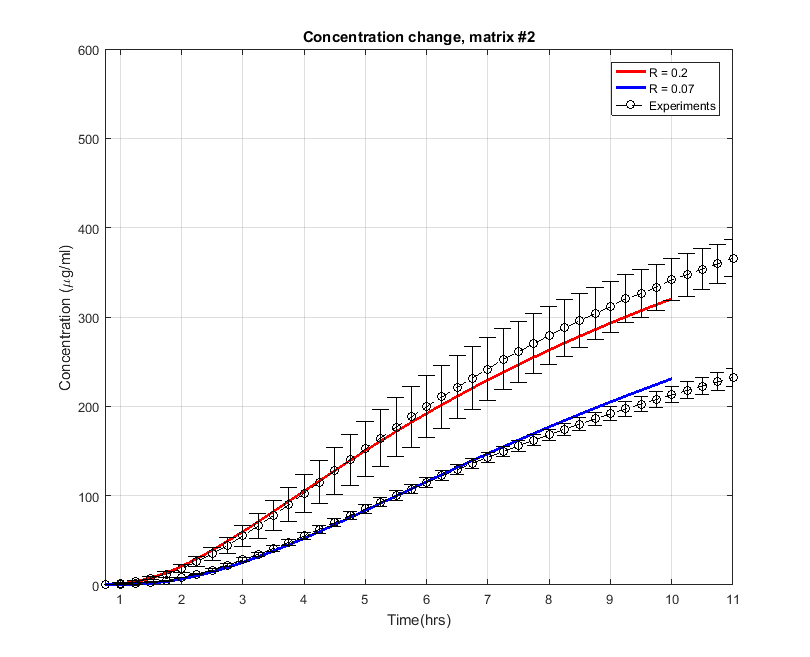

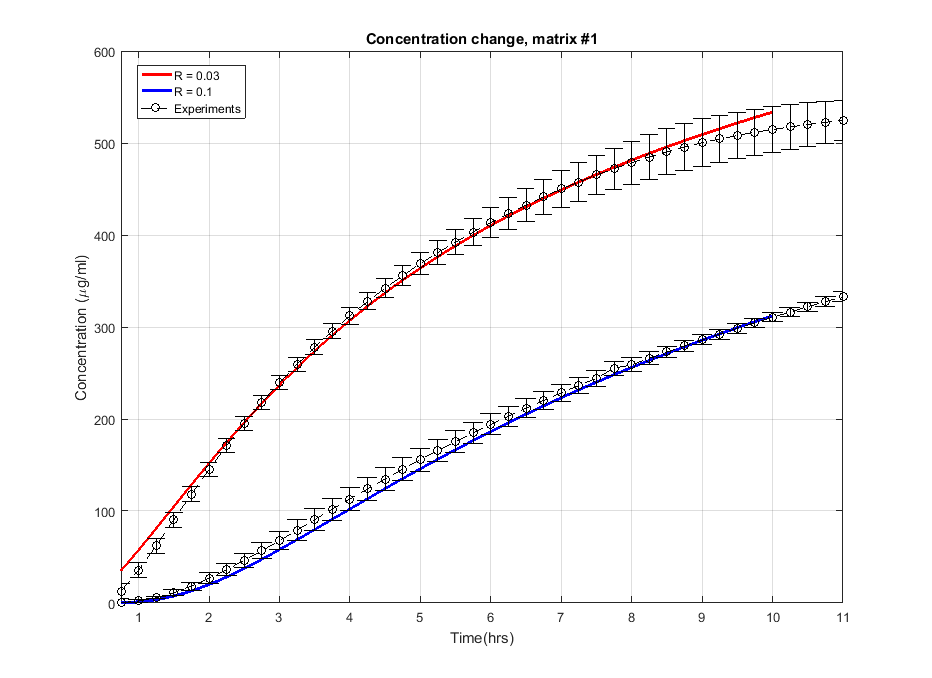


(a)

(b)

**Fig. s3** Concentration of the chemoattractant in the top channel vs. time **on the edge** (NOT center) **of** matrix #1 (a) and #2 (b) for two different chips. R is the ratio of the diffusion coefficient of the chemoattractant (EGF) in the matrix and in water. The simulations are done in COMSOL Multiphysics and the experiments are the result of diffusion of fluorescent dextran from the bottom channel through the matrix to the top channel.


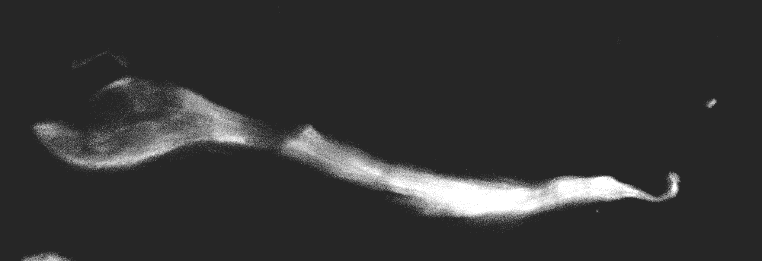


**Fig. s5** Immunofluorescence image of an MDA-MB-231 breast cancer cell inside a PCL electrospun matrix. The cells are stained for F-actin (Alexa Fluor 488). The image is a Z projected image of multiple confocal image slices 3 µm apart. The scale bar is 10 µm.


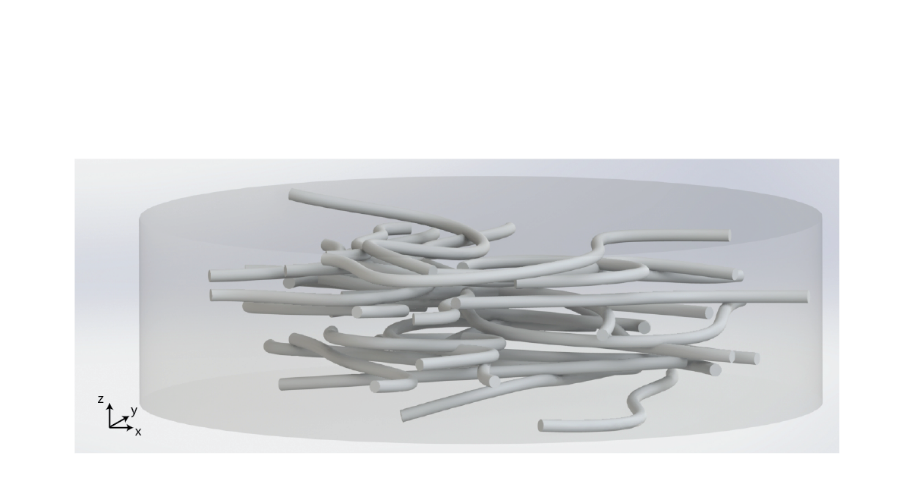


**Fig. s6** A schematic of intrinsic alignment of fibers in the electrospinning process. The fibers are deposited on the collector in the x-y plane, and thus mostly form a 90° angle with the z axis. In our microfluidic system, the chemoattractant gradient and potentially the net migration is along the z axis. Therefore, the intrinsic fiber orientation can act against migrating cells. This image is an impression of the observed fluorescence and scanning electron microscopy micrographs of electrospun matrices.

**Fig. s7** Microfluidic chip used in this study. Two Electrospun matrix layers are integrated between two microfluidic channels. Female luer adapters (without the tubing connection end) were glued to the inlets and outlets as reservoirs.


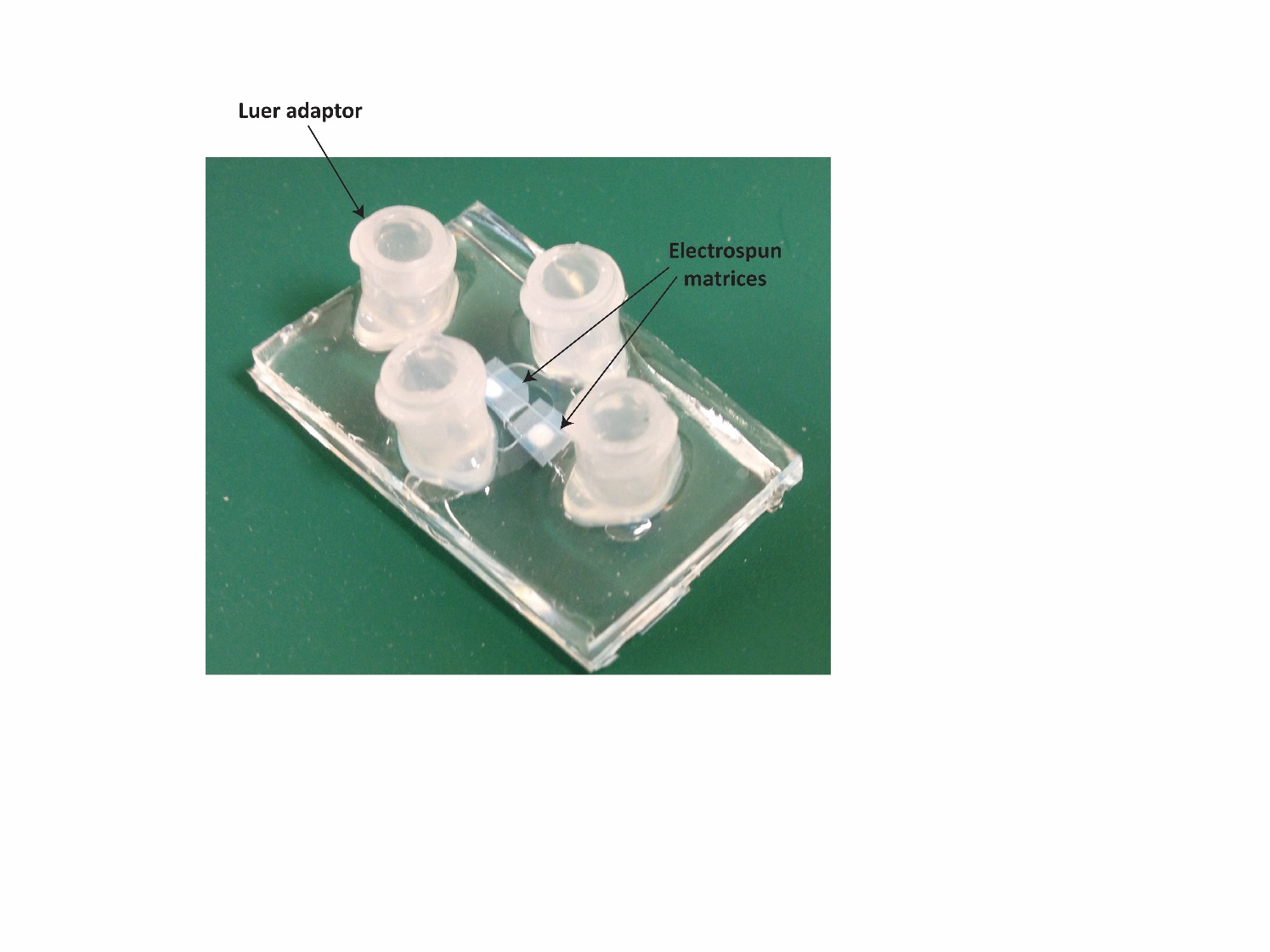


1. H. Eslami Amirabadi **∙** S. SahebAli **∙** J.P. Frimat **∙** R. Luttge **∙** J.M.J. den Toonder, [j.m.j.d.toonder@tue.nl](mailto:j.m.j.d.toonder@tue.nl) (🖂), +31402475706 (🕿), Microsystems group, Department of Mechanical Engineering and Institute for Complex Molecular systems (ICMS), Eindhoven University of Technology, Groene Loper 15, 5612AZ Eindhoven, the Netherlands. [↑](#footnote-ref-1)
